# Upstream regulatory architecture of rice genes: summarizing the baseline towards genus-wide comparative analysis of regulatory networks and allele mining

**DOI:** 10.1186/s12284-015-0041-x

**Published:** 2015-02-28

**Authors:** Benildo G de los Reyes, Bijayalaxmi Mohanty, Song Joong Yun, Myoung-Ryoul Park, Dong-Yup Lee

**Affiliations:** School of Biology and Ecology, University of Maine, Orono, ME 04469 USA; Department of Chemical and Biomolecular Engineering, National University of Singapore, Singapore, 117576 Singapore; Department of Crop Science and Institute of Agricultural Science and Technology, Chonbuk National University, Chonju, 561-756 Korea

**Keywords:** Cis-elements, Trans-acting factors, Spatio-temporal regulation, Regulatory network, Comparative genomics

## Abstract

Dissecting the upstream regulatory architecture of rice genes and their cognate regulator proteins is at the core of network biology and its applications to comparative functional genomics. With the rapidly advancing comparative genomics resources in the genus *Oryza*, a reference genome annotation that defines the various cis-elements and trans-acting factors that interface each gene locus with various intrinsic and extrinsic signals for growth, development, reproduction and adaptation must be established to facilitate the understanding of phenotypic variation in the context of regulatory networks. Such information is also important to establish the foundation for mining non-coding sequence variation that defines novel alleles and epialleles across the enormous phenotypic diversity represented in rice germplasm. This review presents a synthesis of the state of knowledge and consensus trends regarding the various cis-acting and trans-acting components that define spatio-temporal regulation of rice genes based on representative examples from both foundational studies in other model and non-model plants, and more recent studies in rice. The goal is to summarize the baseline for systematic upstream sequence annotation of the rapidly advancing genome sequence resources in *Oryza* in preparation for genus-wide functional genomics. Perspectives on the potential applications of such information for gene discovery, network engineering and genomics-enabled rice breeding are also discussed.

## Introduction

The ‘gene-centric’ and subsequently the ‘genome-enabled’ paradigms of functional biology have led to concerted efforts to understand the biochemical synergies in the plant cell, by examining how genes function in a spatio-temporal scale. Understanding the multi-layered complexity of gene regulation is central to this goal, from the process of chromatin remodeling to transcription initiation, post-transcriptional processing and modulation, and protein modification and interaction. The process of transcription initiation is a critical crossroad of such multi-layered complexity of gene regulation, because it is an early step in the process where coordination could be interfaced with various intrinsic and extrinsic signals for growth, development, reproduction and adaptation. Thus, the study of the architecture of promoters and the nature of the interacting general and specialized regulatory transcription factors has always been an important aspect of understanding the intricate wirings of gene function (Kaufmann et al., 2010; Mejia-Guerra et al., 2012).

A large number of rice genes of potential agronomic value are regulated largely at the transcriptional level. Looking back to as early as the mid-1980’s, rice geneticists and molecular biologists have always been interested in studying the architecture of promoters, initially for the primary objective of discovering regulatory sequences for spatio-temporal engineering of transgenes. Examples of seminal studies include the dissection of the regulatory elements for precise endosperm-specific expression of seed storage proteins, which also elucidated the mechanisms of how seed development and maturation are regulated, and led to applications in seed bioengineering (Takaiwa et al., 1987; Okita et al., 1989; Takaiwa et al., 1991).

Subsequently, the elucidation of the entire genome of a reference genotype of cultivated rice (*O. sativa* ssp. japonica, cv. Nipponbare) made the establishment of direct links between agronomically important physiological processes and gene regulation at the whole transcriptome level possible (Ohyanagi et al., 2006; Yun et al., 2010; Sato et al., 2011; Hamada et al., 2011). The enormous amount of transcriptome data generated to date has routinely spark the same old question that rice geneticists has long been trying to address even prior to the advent of the genome-enabled era of biology, that is the question of: *What unifying DNA signals determine the spatio-temporal transcription patterns of rice genes* and *how do these elements work?* In today’s context, we often ask a more elaborate question of: *What cis*-*elements and trans-acting factors facilitate the coordinate expression of functionally related rice genes and what can we learn from such synergy to understand the network relationships among genes in a grander scale that can explain phenotypic variation?* As we get more advanced in the era of integrative, predictive and comparative systems biology in rice research, such a question has become quite relevant in the context of subsequent translation of genomics to various applications in molecular breeding, allele mining and network engineering for the improvement of yield and nutritional value of rice.

This review addresses a fundamental question that is central to the understanding of how transcription of rice genes is programmed. Its primary motivation is the need to present a synthesis of the current state of knowledge and consensus trends regarding the cis-acting and trans-acting components of transcriptional regulatory modules using representative examples from both seminal and more recent studies. It is hoped that this review will catalyze concerted efforts to continuously compile and update information in the same dynamic pace as in the dicot model Arabidopsis. While the information contained in this review is by no means inclusive of the entire body of information available about this topic, it is to our knowledge the first attempt to integrate the results of seminal studies with more recent updates about the structure, organization and evolutionary conservation of cis-elements in rice. Integration of this body of knowledge should nucleate new concepts on the application of regulatory sequences to comparative genomics, allele mining, and genetic manipulation by network engineering.

## Review

### Core promoter architecture and basal transcription machinery

With the annotated reference genome sequence of *O. sativa ssp. japonica* (cv. Nipponbare) and a rapidly increasing volume of whole-genome resequencing data on representative germplasm diversity panel across the genus, one important feature that needs to be defined for a biologically meaningful interrogation of protein-coding genes is the proximal regulatory region or ‘*core promoter*’. The core promoter is at the very heart of the process of transcription, being the site for the assembly of various protein components of RNA polymerase II pre-initiation complex, and its cooperation with other cis-elements and trans-acting factors that facilitate precise spatio-temporal regulation.

**Figure 1:**
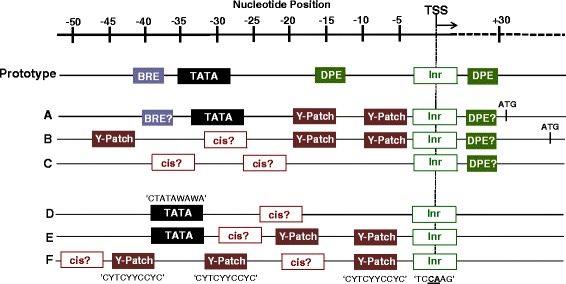
**Comparison of the core promoter architectures of rice genes with the prototype core promoters of metazoan and Arabidopsis genes.** In addition to the *TATA-box*, the composite prototype model of metazoan core promoter shows all previously identified cis-elements (*BRE*= TFIIB recognition element; *DPE*= Downstream promoter element; *Inr*= Initiator sequence) occurring in various combinations with the *TATA-box*. In Arabidopsis genes **(A,** **B,** **C)**, *TATA-box*-containing core promoters **(A)** represent about 30% of all the protein-coding genes encoded by the genome. Non-TATA-box-containing promoters **(B,** **C)** represent a larger proportion of Arabidopsis genes. Small groups of genes contain novel motifs (cis?) that appear to be specific to higher plants. Evidence of the importance of *DPE-like* and/or *BRE-like* sequences in the functionality of Arabidopsis core promoters have not been established so far. In rice **(D,** **E,** **F)**, *TATA-box*-containing core promoters **(D)** represent only about 18% of all protein-coding genes in the rice genome. *Y-Patch* is found in as much as 50% of the total protein-coding genes either in combination with or independent of *TATA-box* **(E,** **F)**. *DPE-like* and *BRE-like* sequences are insignificantly represented in the core promoters of rice genes.

So what constitutes a core promoter? The consensus core promoter of eukaryotic genes is a contiguous stretch of DNA comprised of various cis-acting elements (Figure 1). The most well characterized is the *TATA-box*, defined by the consensus sequence ‘TATAWAWAR’, where the upstream ‘T’ is often located around −30 relative to A +1 (or G +1) position of the initiator (*Inr*) sequence for the transcription start site or TSS (Smale and Baltimore, 1989). The *TATA-box* is recognized by a sub-unit of the general transcription factor TFIID called the TATA-binding protein (TBP) to facilitate the formation of pre-initiation complex (Butler and Kadonaga, 2001; Smale, 2001). Occasionally found upstream to the *TATA-box* is a less frequently occurring motif called *BRE* (TFIIB recognition element), which facilitates efficient binding of transcription factor TFIIB to the core promoter. About 30-nt downstream of *Inr* is the Downstream Promoter Element (*DPE*), which is recognized by the TBP-associated factor (TAF) to enhance TBP binding to the core promoter (Burke and Kadonaga, 1997). In some cases *TATA-box*, *Inr* and *DPE* all occur in the same promoter (Juven-Gershon and Kadonaga, 2010).

Structural conservation of TFIID in metazoans and plants suggests that the cis-acting elements involved in RNA polymerase II-dependent transcription are similar among eukaryotes (Vogel et al., 1993; Smale, 2001). Pioneering studies on the rice phenylalanine ammonia-lyase (*PAL*) promoter *ZB8* showed that 5’-truncation up to −35 could maintain an accurate initiation of basal transcription (Zhu et al., 1995). The ‘TATTTAA’ motif within −35 to −28 that resembles the consensus sequence for eukaryotic *TATA-box* is critical for minimal promoter activity. Replacement of this sequence either with G-rich motif (‘GCGGGTT’) or with 2-nt substituted versions (T***CG***TTAA or TAT***GG***AA) could lead to complete loss of promoter function. It has been shown that a recombinant TBP of rice (*OsTBP2*) interacts with *OsTFIIB* for efficient binding to the *TATA-box* in *ZB8* promoter, causing highly efficient TATA-dependent basal transcription (Zhu et al., 2002). Moreover, the functionality of −35 to −28 region of *ZB8* is in context with the consensus ‘TC***CA***AG’ *Inr* sequence. Substitution of the conserved core ‘CA’ motif at −1 and +1 positions of *Inr* leads to erroneous initiation at multiple locations.

Contrary to earlier notions that *TATA-box* is a universal feature of most eukaryotic core promoters, subsequent surveys of Arabidopsis, human and Drosophila genomes have shown that this was not the case (Molina and Grotewold, 2005; Lenhard et al., 2012). For instance, although the *TATA-boxes* among Arabidopsis genes were contextually positioned at the correct upstream location, their occurrence in such context was established only in less than 30% of the total validated genes. While the occurrence of *TATA*-related motifs was established reliably among highly expressed genes, other more prominent sequence motifs distinct from *BRE* and *DPE* have been identified in smaller subsets of TATA-less Arabidopsis genes (Figure 1).

Indeed, more recent surveys of *TATA-box* distribution in the rice genome established a trend similar to what has been established in Arabidopsis (Figure 1). Independent analysis with Gibbs Motif Sampler and Dragon Motif Builder showed that less than 20% of annotated rice genes contain *TATA box*-like motifs in the right context. Furthermore, *DPE*-like and/or *BRE*-like sequences are not significantly represented in rice core promoters (Clivan and Svec, 2009). The consensus sequence for rice *TATA-box* is defined as ‘CTATAWAWA’ located within a C-rich region around −49 to −20. However, much higher occurrence and broader distribution of a T/C-rich motif called *Y-Patch* (pyrimidine patch) was established in a global survey of rice core promoters. Although it has lesser degree of sequence conservation than *TATA-box*, *Y-Patch* elements occur in correct upstream context in more than 50% of annotated rice genes, either in combination with or independent of *TATA-box*. The consensus *Y-Patch* sequence has been defined as ‘CYTCYYCCYC’, occurring in one or more copies and with strict directional sensitivity around −49 to +1 region (Yamamoto et al., 2007; Clivan and Svec, 2009).

### Key regulatory modules for constitutive expression of rice genes

**Figure 2:**
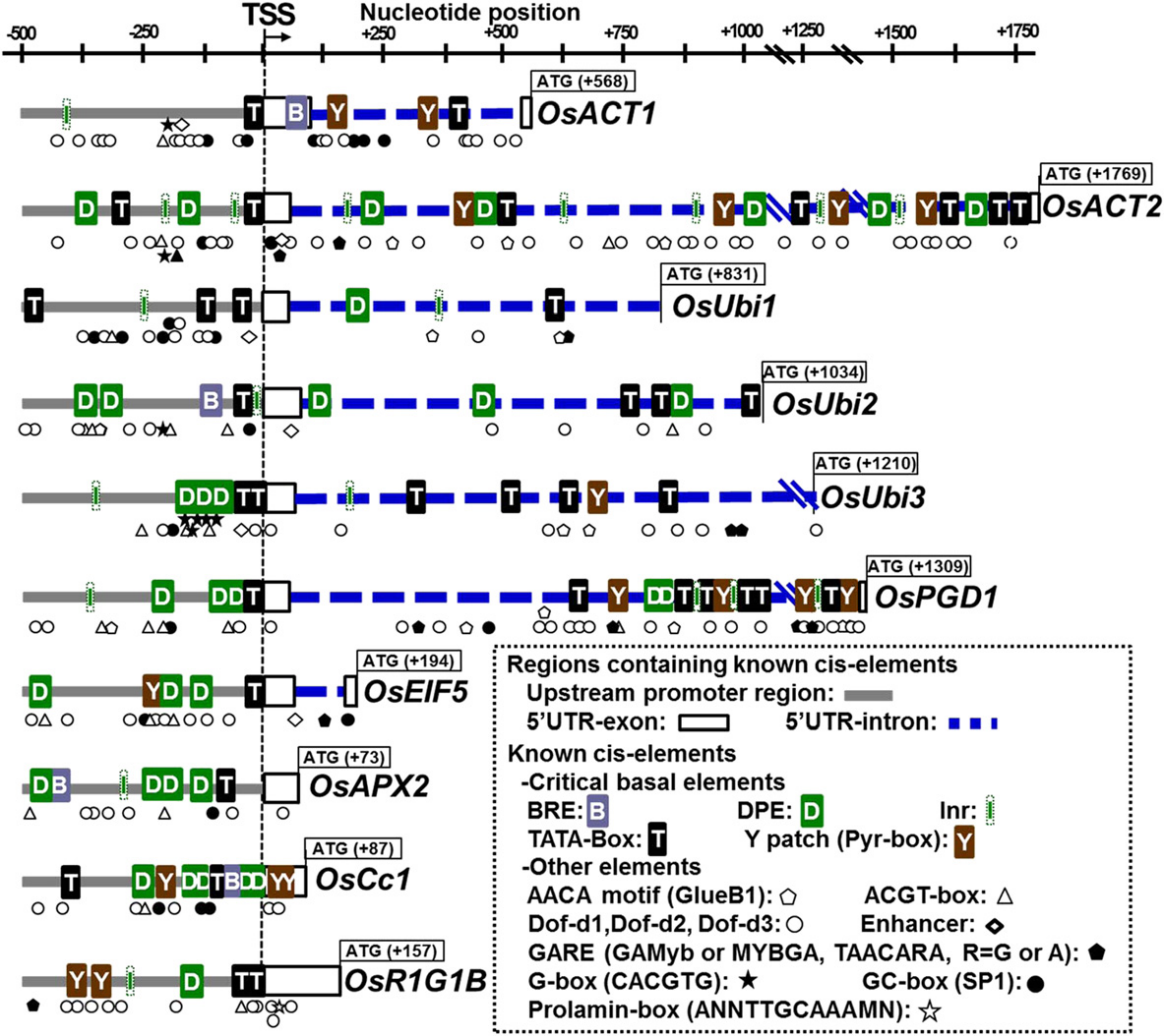
**Models of the regulatory sequence architectures of well characterized promoters of constitutively expressed rice genes such as actin (** ***OsACT1, OsACT2*** **), ubiquitin (** ***OsUbi1, OsUbi2, OsUbi3*** **), phosphogluconate dehydrogenase (** ***OsPGD1*** **), Initiation factor (** ***OsEIF5*** **), ascorbic peroxidase (** ***OsAPX2*** **), cytochrome-c (** ***OsCc1*** **), and R1G1-domain-containing protein (** ***OsR1G1B*** **).** Locations of critical cis-elements are indicated with colored symbols in both the upstream regions (gray lines) and downstream regions (5’UTR-exon, 5’UTR-intron; blue lines) relative to the location of the transcription start site (TSS).

A number of constitutive promoters have been particularly well investigated in rice including actin (*OsAct*), ubiquitin (*OsUbi*), cytochrome-c (*OsCc1*), ascorbate peroxidase (*OsAPX*), translation initiation factor-5 (*OseIF5*), phosphogluconate dehydrogenase (*OsPGD*), and R1G1-domain containing protein B (*OsR1G1B*) genes. The shared structural feature of these promoters is a short exon and large intron in the untranslated region (UTR) near the TSS, in addition to upstream regulatory modules (Figure 2, Table 1). The 5’-UTR introns are A/T-rich but share little sequence identity and with variable lengths (Jeong et al., 2006).

**Table 1:** **List of experimentally or computationally defined upstream regulatory sequences, and their cognate transcriptional regulators that determine basal and constitutive expression of rice genes**

**Cis-element**	**Core motif**	**Transcriptional regulator**	**Biological function**
***TATA-box***	CTATAWAWA	TATA-binding protein (*OsTBP2*)	Core promoter; Pre-initiation complex
***Y-Patch***	CYTCYYCCYC	Unknown	Core promoter; Pre-initiation complex
(Pyrimidine patch)			
***Actin (Act) elements***	CCCAA (tandem octa-repeats)	Unknown	Fine-tuner of constitutive *OsAct* genes
	CAAT (CCAT-box)	Unknown	Positive regulator of constitutive *OsAct* genes
	GTGAC (intronic)	Unknown	Positive regulator of constitutive *OsAct* genes
	CCGCGTTGGC (ABA-responsive)	Unknown	Positive regulator of constitutive *OsAct* genes
	ATTAAT, CACGTA (light-responsive)	Unknown	Positive regulator of constitutive *OsAct* genes
	(A/T)GCC (dideca-repeats)	Unknown	Positive regulator of constitutive *OsAct* genes
***Ubiquitin (Ubi) elements***	GTTGTGGTTTG	Unknown	Positive regulator of constitutive *OsUbi* genes

List was based on seminal studies published in the literature and annotated in public databases.

The strong inducing effects of *OsAct* and *OsUbi* promoters are to a large degree due to the UTR introns. Intron-mediated enhancement (*IME*) of transcription requires the transcribed sequences near the TSS in the right orientation. In general, *IME*-type introns in rice are longer than non-enhancing introns and contain more regulatory elements (Bradnam and Korf, 2008; Parra et al., 2011). Dispersed within the *IME-*type intron are potential cis-elements including the predominant pentamer motif ‘CGATT’. It has been suggested that *IME*-type introns act through gene loops that facilitate steady reinitiation of transcription by RNA Pol II (Moabbi et al., 2011; Rose et al., 2011). The coding sequences of ubiquitin (*OsUbi3*) monomer often involving the first nine nucleotides enhance promoter activity over 4-fold (Clancy et al., 1994). In addition, the most commonly found elements in the 5’ upstream and UTR intron are Dof, GC-box, and ACGT-box (Figure 2, Table 1).

Typical cis-elements in canonical core promoters are also important elements required for strong constitutive expression of *OsAct*. For example, *OsAct1* promoter contains a *TATA-box* at −35, a *BRE-like* motif at position +88 and two copies of *Y-Patch* element at positions +138 and +384. In addition, the region from position −50 are highly enriched with other potential regulatory elements including *Sp1*, light-responsive *SORLIP*, mesophyll-specific *CACTFTPPCA1*, *Alfin1*, a novel zinc-finger binding element found in the salt stress inducible genes, a signature element of nitrate reductase gene *AMMORESIIUDCRNIA1* and a drought-responsive *SBOXATRBCS* (Figure 2, Table 1).

The critical regulatory elements of rice actin genes (*OsAct1*, *OsAct2*) include a 5’-upstream sequence, non-coding exon, 5’-UTR region intron, and a short non-coding region of the second exon (Figure 2). The first non-coding exon and intron are 79-bp and 58-bp, and 447-bp and 1,736bp for *OsAct1* and *OsAct2*, respectively (McElroy et al., 1991; Zhang et al., 1991; He et al., 2009). Many cis-elements required for constitutive expression are located around the core promoter region, including a 38-bp poly(dA-dT) between positions −146 and −186 which act as a positive regulator, and eight tandemly repeated copies of imperfect pentamer motif with consensus ‘CCCAA’ at −294, acting as a negative regulator. In addition, the branch-point site (‘GTGAC’) for mRNA splicing located in the intron between the positions 359 and 363 is essential for promoter activity (McElroy et al., 1990).

A negative regulator between positions +96 and +274 within the intron is responsible for the repression of *OsAct2* expression. On the other hand, the two *CAAT-boxes* between positions −448 and −445 and positions −635 and −632 act as possible positive regulators. Other cis-elements in the 5’-upstream region include ABA-responsive element (‘CCGCGTTGGC’), two different light responsive elements (‘ATTAAT’, ‘CACGTA’), and 20 repeated ‘A/TGCC’ triplets. Numerous cis-elements are also present between positions −50 and TSS, including *CAATBOX1, SORLIP1AT, Alfin1, GATABOX, UPRMOTIFIIAT, C-rich Q, BOXCPSAS1*, and ‘CGTGG’ motif.

Ubiquitin (*OsUbi*) promoter includes the 5’-upstream sequence and 5’-UTR containing a short first exon and intron (Wang and Oard, 2003; Bhattacharyya et al., 2012). Critical elements include a signature enhancer motif (‘GTTGTGGTTTG’) found in both *OsUbi1* and *OsUbi2* (Christensen and Quail, 1996; Wang and Oard, 2003). In addition to a *TATA-box*-like element, core promoters of *OsUbi* contain other signature cis-elements associated with inducible expression such as *Alfin1*, *Dof*, *CARGCW8GAT*, *TAAAGSTKST1*, *PRECONSCRHSP70A*, *GT-box*, *POLLEN1LELAT52*, *WUSATAg*, and *ARR1AT* (Figure 2, Table 1).

The promoter of cytochrome-c (*OsCc1*) includes the 5’-upstream region and 86-bp untranslated sequence without UTR intron (Figure 2). In addition to the signature core promoter elements such as TATA-box and CAAT box, it contains the cAMP response element (CRE), nuclear respiratory factor (NRF) binding site, and light responsive G-box (CACGTV) (Jang et al., 2002). Promoters of ascorbate peroxidase (*OsAPX2*), phosphoguconate dehydrogenase (*OsPGD1*), and *R1G1*-domain-containing protein (*OsR1G1B*) have similar constitutive strengths as the promoter of *OsAct1*, with various enhancers such as light responsive G-box and TGACG-element associated with high-level gene expression. Endosperm-specific regulatory elements such as ‘AACA’ and ‘ACGT’ are also important characteristics of *OsPGD1* and *OsR1G1B* (Park et al., 2010a; Figure 2, Table 1). Based on the trends established from the analysis of housekeeping genes, it is apparent that constitutive expression requires many of the elements of the core promoter in various combinatorial complexities with various UTR structural features and a plethora of cis-elements involved in developmental and environmental signal perception.

### Integration of hormonal, developmental and stress response signaling

Plant growth, development and adaptation are consequences of cooperative and antagonistic actions of different regulatory hormones, starting from germination to the development and maturation of seeds. Integration of hormonal and other intrinsic developmental and environmental signals leads to changes in gene expression.

#### Root development and auxin signaling

Auxin is recognized as the universal plant growth hormone because of its principal role in the regulation of cell expansion, division and differentiation (Kieffer et al., 2010). The principal receptor of auxin is a class of nuclear F-box protein that belongs to the TIR1/AFB family (Darmasiri et al., 2005). Auxin perception leads to rapid induction of three classes of auxin early responsive genes, which include the *Aux/IAA*, *GH3* and the small auxin-up RNA or *SAUR* (Hagen and Guilfoyle, 2002). *Aux/IAA* regulates its own transcription, and functions as transcriptional regulator of other auxin responsive genes in cooperation with Auxin Response Factors (ARF) (Chapman and Estelle, 2009). ARF binds specifically to the promoters of *GH3*, *SAUR* and other auxin-responsive genes leading to rapid induction (Ulmasov et al., 1995; Ulmasov et al., 1997). In the absence of auxin, Aux/IAA inhibits ARF function and represses other auxin early responsive genes. Auxin binding activates TIR1/AFB, a component of ubiquitin E3 ligase SCF1, mediating ubiquitination and degradation of Aux/IAA. In effect ARF is relieved from repression, hence expression of auxin early responsive genes (Tiwari et al., 2003; Quint and Gray, 2006). In rice, 25 *OsARF* homologs have been identified, indicating a high degree of conservation across the dicot and monocot lineages (Wang et al., 2007).

Analysis of the japonica rice reference genome identified 31, 12 and 58 members of the *OsAux/IAA*, *OsGH3* and *OsSAUR* gene families, respectively, many of which exhibit the characteristic rapid induction in response to auxin (Thakur et al., 2001; Jain et al., 2006a; Jain et al., 2006b, Jain et al., 2006c; Song et al., 2009). Moreover, it has been estimated that between 2% to 5% of the total genes in Arabidopsis and rice are either upregulated or downregulated by auxin (Nemhauser et al., 2004). The signature of the promoter of auxin early responsive genes is a 6-bp motif with the consensus sequence ‘TGTCTC’, often referred to as auxin-response element (*AuxRE*), occurring in either simple or composite configurations (Ulmasov et al., 1997). Simple *AuxR*E contains either direct or palindromic repeats of ‘TGTCTC’ motif separated by 3 to 7-nt spacer with no apparent consensus sequence. In contrast, composite *AuxRE* is functional only in modular form with CG-rich ‘coupling’ or ‘constitutive’ element (Table 2).

**Table 2:** **List of experimentally and computationally defined upstream regulatory sequences, and their cognate transcriptional regulators that determine spatio-temporal control of rice genes**

**Cis-element**	**Consensus core motif**	**Transcriptional regulator**	**Biological function**
***Auxin responseelement (AuxRE1)***	TGTCTC	Auxin responsefactor (*ARF1, ARF2*)	Primary regulator of auxin-regulated expression
***Auxin responseelement (AuxRE2)***	GACACA	Auxin responsefactor (*ARF1, ARF2*)	Primary regulator of auxin-regulated expression
***Auxin responseelement (AuxRE3)***	GACActGACA	Auxin responsefactor (*ARF1*)	Primary regulator of auxin-regulated expression
***Gibberellin responseelement (GARE)***	TAACCACC	*GAMyb* factors	Primary regulator of GA-regulated expression; primary regulator of sugar sensitivity of GA-responsive genes
***Glut element***	AACA/ACGT	Unknown	Regulator of endosperm-specific expression of rice seed storage protein glutelin (*OsGlut)*
***Gt1/Gt2 box-I***	ATATCATGAGTCACTTCA	*GT1, GT2*	Positive regulator of endosperm-specific expression of rice glutelin (*OsGlut*) genes
***Gt1/Gt2 box-II***	CTTTCGTGTACCACA	*GT1, GT2*	Positive regulator of endosperm-specific expression of rice glutelin (*OsGlut*) genes
***Gt1/Gt2 box-III***	ACAATGCTGCTCAATTA	*GT1, GT2*	Positive regulator of endosperm-specific expression of rice glutelin (*OsGlut*) genes
***Gt1/Gt2 box-IV***	ATTATCCAATGTCATATTG	*GT1/GT2*	Positive regulator of endosperm-specific expression of rice glutelin (*OsGlut*) genes
***Gt1/Gt2 box-V***	TAAGTCACGTTTGATGA	*GT1, GT2*	Positive regulator of endosperm-specific expression of rice glutelin (*OsGlut*) genes
***Gt3 box***	ATATCATGAGTCACTTCA	*GT1, GT2, GT3*	Positive regulator of endosperm-specific expression of rice glutelin (*OsGlut*) genes
***PB-1 element***	AAGCAACACACAAC	Dof-type P-boxBinding factor (*PBF*)	Positive regulator of endosperm-specific expression
***Prolamin box***	TGTAGAA, TGTTTTAATATGACGTGG	Dof-type P-boxbinding factor (*PBF*)	Positive regulator of endosperm-specific expression
***SP8/SURE***	TACTATT, TCACTATT,	*Opaque-2* transcriptionfactor	Positive regulator of endosperm-specific expression
***LPSE1***	ATTTGAGCTGCC	Unknown	Positive spatial regulator of expression in green leaves, stem and young panicle
***LPSE2***	TTGATATATTTGT	Unknown	Positive regulator of expression on green leaves and negative regulator of expression in young panicle and roots
***LPSRE1***	CGGCGCGCCAC	Unknown	Positive regulator of expression in green leaves and negative regulator of expression in panicle and stem
***LPSRE2***	TTAGATAATGGA	Unknown	Positive spatial regulator of expression in green leaves, stem root, and young panicles
***PSE1***	TTTATCTATTTCC	Unknown	Negative regulator of expression in young panicle and stem
***PSE2***	TCTTTGGCAGAG	Unknown	Positive regulator of expression in green leaves and negative regulator of expression in stem and young panicles
***Ethylene and jasmonic acid responsive element***	GCC-CORE	Ethylene response factor	Regulator of senescence-induced gene expression (*OsERF*)
***GS2***	TTCGAAATTGAACGTGCT-TAACCAAGAGAACAC	*WRKY* transcription factors	Senescence box
***ABRE (ACGT-type)***	CGMCACGTB	*OsABI5, OREB1*	ABA-response element for ABA regulated gene expression during stress response and seed maturation
***CE1***	TGCCACCGG	*OsABI5, OREB1*	ABRE coupling factor
***CE3***	ACGCGTGTCCTC	*OsABI5, OREB1*	ABRE coupling factor
***DRT/CRE***	(a/g)CCGAC	*OsDREB1, OsDREB2*	Dehydration response element for abiotic stress regulated gene expression independent of ABA
***Abiotic/oxidative stress Element***	CACG	*OsNAC6, OsNAC5*, *OsSNAC2*	Regulator of abiotic stress-responsive gene expression independent of the DREB regulon
***as1/ocs/TGA-like element***	AATTTGAT, TAATTTGA	*OsTGA10*	Regulator of chilling mediated oxidative stress signaling
***Pyrimidine-box/Myb2-box element***	AAAGAAAAA, TAGTTTTT	*OsMyb4*	Regulator of chilling mediated oxidative stress signaling
***JARE***	CGTCA, TGACG, VCGCGB	Ethylene response factors (*ERF*)	Jasmonic acid response element; regulator of responses to pathogens and herbivores
***GCC-box***	TAAGAGCCGCC	Ethylene response factors (*ERF*)	Negative regulator of responses to *Magnaporthe oryzae*
***P-box***	CCGCCCTCCA	Ethylene response factors (*ERF*)	Disease responsive element
***W-box***	TTGAC element	*OsWRKY45, OsWRKY71*, *OsWRKY03, OsWRKY53*, *OsWRKY31, OsWRKY30*, OsWRKY76	Disease responsive
***E-box***	CAGTCG, CACCTG	*OsbHLH65*	Pathogen and herbivore response element; regulator of responses to *Magnaporthe Oryzae* and *Nilaparvata lugens*

List was based on seminal studies published in the literature and annotated in public databases.

The analysis of the *crown rootless* (*crl1*) mutant, defective in auxin-mediated formation of crown roots facilitated the elucidation of cis-elements and trans-acting factors involved in auxin-regulated gene expression in rice (Inukai et al., 2005; Liu et al., 2005a). The *crl1* mutation exhibits various auxin-related abnormalities including reduced lateral roots, insensitivity to auxin during lateral root initiation, and impaired root gravitropism. Wild-type *Crl1* encodes an Assymetric leaves-2/Lateral Organ Boundaries (AS2/LOB) domain protein containing the N-terminus C-motif and GAS-motif at the C-terminus. The mutant protein contains a single amino acid substitution (A to T) within the AS2/LOB domain. The wild-type *Crl1* is strongly induced by auxin, dependent on the degradation of Aux/IAA proteins, as shown by the effects of mutation in the degradation domain (domain II) of *OsIAA3*. The fact that Aux/IAA functions as a repressor of the transcriptional activator ARF has implied that *Crl1* is a direct target of *OsARF* transcription factors. The promoter of wild-type *Crl1* has two copies of *AuxRE* signature sequence. The first copy (*AuxRE1*) located upstream and distal from the *TATA-box* has the ‘TGTCTC’ sequence, while the second copy (*AuxRE2*) located upstream but more proximal to the *TATA-box* has the inverted sequence ‘GACACA’ (Table 2). The primary auxin-response transcriptional activator in rice (*OsARF1*) binds with high affinity with *AuxRE2* but not with *AuxRE1* (Waller et al., 2002).

Another *crown rootless* mutant in rice (*crl5*) has impaired initiation of crown root primordia (Kitomi et al., 2011). Mutants have abnormal root gravitropic response, delayed flowering, various types of floral anatomical abnormalities and small panicles. In wild-type plants, *Crl5* is rapidly induced by exogenous auxin in the nodes or in regions of the stem where root growth is initiated. Like *Crl1*, rapid expression of *Crl5* is also dependent upon the degradation of Aux/IAA proteins, thus *Crl5* is downstream to *OsAux/IAA* and *OsARF*. The 642-aa residue CRL5 protein contains two copies of AP2 DNA-binding domain, related to AP2/ERF transcription factor *AINTEGUMENTA (ANT)*. The promoter of *Crl5* contains three copies of the ‘TGTC’ core motif of *AuxRE*. The first copy (*RE1*), which is upstream and most distal to the *TATA-box* consists of a tandem array of three inverted ‘TGTC’ motifs (‘GACAGACAGACA’). The second copy (*RE2*) is a single base substituted version (‘TGTCGC’) of the consensus for AuxRE. The third copy (*RE3*), which is upstream but most proximal to the *TATA-box* has two tandem ‘TGTC’ motifs with a dinucleotide spacer in between, i.e., ‘GACActGACA’ (Table 2). Direct binding of recombinant *OsARF1* was demonstrated for the most proximal wild-type *RE3* but not for its mutated version ‘GATActGATAct’, indicating that *Crl5* is directly activated by *OsARF1 like Crl1*. The consensus from the analysis of *Crl1* and *Crl5* is that integration of auxin-responsive gene expression during root development requires a proximal *AuxRE*.

#### Seed germination and gibberellic acid signaling

First discovered in rice, the signature effect of gibberellic acid (GA) is the ability to promote stem elongation. Bioactive GAs affect virtually all aspects of plant growth and development from germination to hypocotyl elongation, stem growth, circadian rhythm, and reproductive organ and seed development (Lovegrove and Hooley, 2000; Hartweck and Olszewski, 2006).

Discovered first in rice, *GID1/GID2* genes encode the nuclear localized primary receptor of GA (Gomi et al., 2004; Ueguchi-Tanaka et al., 2005; Griffiths et al., 2006; Ueguchi-Tanaka et al., 2007). Another important component of GA signaling are the DELLA proteins encoded by *SLR1* in rice and *GAI/RGA-like* genes in Arabidopsis, which function as negative regulators of GA response (Ikeda et al., 2001; Fleet and Sun, 2005; Itoh et al., 2005). The rice DELLA protein is stable in the absence of GA ensuring that transcription of GA-early responsive genes are repressed by blocking the activity of a transcriptional activator bound to the promoters of target genes. GA binds to the nuclear localized GID1 receptor, triggering its interaction with the DELLA repressor. The GID1-DELLA complex recruits the GID2 F-box protein, a component of the SCF ubiquitin ligase complex, resulting in the degradation of the DELLA repressor and subsequent transcription of GA-early responsive genes (Hartweck, 2008). Analysis of the GA-response transcriptome estimated that about 250 and 300 genes are regulated by GA in Arabidopsis and rice, respectively (Ogawa et al., 2003; Yazaki et al., 2004).

In rice and other cereals, available sugars in the embryo are rapidly depleted at the onset of germination, triggering synthesis of GA which is then released to the aleurone layer. Subsequent expression of hydrolases ensures sufficient supply of sugar from the breakdown of starch to support seedling growth (Kaneko et al., 2002). Expression of α-amylases during rice germination is repressed by high sugar and abscisic acid (ABA) in the embryo, while they are induced by GA in the endosperm. Dissection of the promoter architecture of α-amylases established a clear picture of the interacting cis-elements that mediate differential regulation (Lu et al., 1998; Chen et al., 2002; Sutoh and Yamauchi, 2003; Chen et al., 2006). Among the members of the germination-associated α-amylase gene family, *αAmy3* (*RAmy3D*) and *αAmy8* (*RAmy3E*) provide an interesting contrast because both genes are synchronously induced by sugar depletion in the embryo, while in the endosperm *αAmy3* is induced only by sugar depletion and *αAmy8* only by GA. Induction of *αAmy8* by GA is also correlated with the expression of *GAMyb* transcription factors.

The sugar response complex (SRC) was identified within the −186 to −82 region of *αAmy3,* comprised of a *GC-box* (~30-nt GC-rich motif) distal to the *TATA-box*, two tandemly repeated copies of *TA-box* (‘TATCCA’) upstream but more proximal to *TATA-box*, and *G-box* (‘CTACGTGG’) in between the two elements. The sugar response complex/GA-response complex (SRC/GARC) of *αAmy8* is located within −318 to −89, comprised of a *GC-box* upstream and distal to *TATA-box*, single copy of proximal *TA-box*, and a gibberellic acid response element *GARE* (‘TAACCACC’) in between the two elements (Table 2). Mutation of *GARE* in the promoter of *αAmy8* confirmed its critical role for GA-regulation as well as sugar-insensitivity (Table 2). Therefore, differential regulation of α-amylases by sugar depletion and GA in embryo and endosperm is facilitated by the interaction of *GARE* with *GC-box* and *TA-box*.

Expression of *OsGAMyb* transcription factors and *αAmy8* are highly coordinated in the endosperm. In rice *gamyb-2* mutant with impaired GA-dependent expression of α-amylases in the endosperm, *αAmy8* becomes non-responsive to GA and repressible by glucose. Similarly, overexpression of *OsGAMyb* transcription factor has no effect on glucose repression of *αAmy3,* while an increase in expression of Os*GAMyb* renders *αAmy8* insensitive to glucose repression. All these results established that *OsGAMyb* is a key regulator allowing differential responses of α-amylase genes to sugar depletion and GA in the endosperm and embryo by virtue of its interaction with *GARE* in *GARC*-promoters (Table 2).

#### Seed storage protein regulation during seed development

Making up as much as 80% of the total endosperm proteins of rice, glutelins serve as major nitrogen and carbon reservoir for the initial energy requirements during germination (Okita et al., 1989). Rice glutelins are classified into three groups: *GluA, GluB,* and *GluC*. The spatio-temporal regulation of three representative members of *GluA* sub-subfamily (*Gt1*, *Gt2*, *Gt3*) have been extensively investigated in an effort to understand their roles in the regulation of seed development. *Gt1* and *Gt2* are transcribed about 5 days after anthesis and continue virtually throughout the entire duration of seed development. In contrast, *Gt3* transcripts accumulate maximally between 5 to 10 days post-anthesis followed by a steady decline (Okita et al., 1989; Zhao et al., 1994).

Promoter studies established that both 5’ distal and proximal cis-elements in *Gt1* promoter are critical for precise spatio-temporal regulation (Zheng et al., 1993). The distal region (−5.1 to −1.8 kb) contains positive elements for temporal regulation and confer as much as 20-fold increase in expression. Leaky expression in leaves, stems, sheaths and roots was observed in promoter-GUS reporter driven by −507 or −154 truncated fragments of *Gt1* promoter. This indicates that while the distal positive element within the −5.1 kb promoter contributes to temporal control of *Gt1*, proximal elements are critical for spatial specification in the endosperm.

DNase-I footprinting identified five protein-binding motifs (box-I to box-V) in *Gt1* promoter ordered sequentially downstream from −434 position: ‘TAAGTCACGTTTGATGA’ (box-V), ‘ATTATCCAATGTCATATTG’ (box-IV), ‘ACAATGCTGCTCAATTA’ (box-III), ‘CTTTCGTGTACCACA’ (box-II), and ‘ATATCATGAGTCACTTCA’ (box-I) (Table 2). Promoter activity is completely abolished when boxes I to IV are mutated simultaneously. Box-II is the most critical, causing about 35-fold reduction in expression when mutated. Consistent with similar spatio-temporal patterns of *Gt1* and *Gt2*, boxes-I to V are highly conserved between the two promoters, except that box-V has varied locations (Kim and Wu, 1990).

Similar to *Gt1*, the functional elements responsible for spatial, temporal and quantitative effects of *Gt3* promoter are located at distal (−945 to −726) and proximal (−346 to −263) regions (Zhao et al., 1994; Croissant-Sych and Okita 1996). The critical elements responsible for precise temporal control post-anthesis are located within +7 to −181 and −278 to −320 regions. Removal of either of these proximal elements causes as much as 50% reduction in expression. The −106 to −88 and −125 to −111 regions of *Gt3* promoter contain sequences similar to box-I and box-II in *Gt1* and *Gt2* promoters, with the same relative positions. Box-I contains the signature binding sequence (‘ATGAc/gTCAT’) for the yeast *GCN4*, *AP-1*, *Jun* and *Fos* transcription factors, while box-II contains another motif found in seed-specific α-amylase genes. *Gt1, Gt2* and *Gt3* promoters also share the signature motif ‘AACA’ around −75 to −64, which is essential for endosperm-specific expression (Table 2).

*Gt3* promoter differed from *Gt1* and *Gt2* promoters by the absence of boxes-III, IV and V. However, its −482 to −278 region contains several functional elements that may be critical for its unique expression including the ‘AAGCAACACACAAC’ motif (−279 to −259) known as the binding site for nuclear factor PB-1, two prolamin-box-like (*P-box*-like) signals ‘TGTAGAA’ (−302 to −295) and ‘TGTTTTAA’ (−322 to −314) known to interact with Dof-type *P-box* binding factor (*PBF*), and ‘TATGACGTGG’ motif, which resembles the consensus binding sequence for the seed-development transcription factor *Opaque-2* (Table 2). *Gt3* promoter also contains additional sequences around −482 region that suppresses its expression in the leaves and other vegetative organs. Studies on other glutelin gene subfamilies also indicated the critical roles of GCN4, AACA-motif and P-box for endosperm-specific expression of *GluB-3* and *GluB-5* (Washida et al., 1999; Qu et al., 2008; Table 2).

#### Tissue/organ-specific gene expression

In rice, the nature of cis-elements and trans-acting factors that confer tissue or organ-specific programs is not yet well understood. However, results of the analysis of few candidate rice genes are starting to reveal interesting trends. One good example is *Oshox1*, encoding a homeo-domain leucine zipper (HD-Zip) transcription factor that plays an important role in vascular differentiation. *Oshox1* expression initiates when procambial cells have already acquired distinctive anatomical properties but before achieving terminal differentiation (Scarpella et al., 2000).

Upstream region (−1,600) of *Oshox1* directs vascular-specific expression through a process that involves auxin and sucrose (Scarpella et al., 2005). *Oshox1* promoter drives expression in a tissue-specific fashion, showing particularly high expression in the apical region of primary and secondary procambial strands of cotyledon, but not in the axis of mature embryo. It also directs expression in the vascular cylinder of the basal mature region of the root, and in the apical hydathode of developing first leaves. Various proximal and distal sections of the −1,600 upstream sequence of *Oshox1* confer different patterns of vascular cell, tissue and organ-specific expression and these sections contain modular combinations of ‘CCA/TTG’ repeats with stretches of ‘CCCC’ or ‘AACA’ motifs (Table 2). The auxin-responsive complex of *Oshox1* promoter is located within −1,621 to −898 region defined by 11 copies of ‘AAGG’ *Dof* element and ‘GGTCCAT’ signature element typical of many known auxin-responsive genes. The same region (−1,621 to −898) is also critical for sucrose-regulated expression, consistent with the occurrence of several known sucrose responsive elements including *SP8 element* (‘TACTATT’), three copies of *SURE* (‘TACTATT’, ‘TCACTATT’, ‘TACTAT’) and one copy of *B-box* (‘CTAAAC’) (Grierson et al., 1994; Ishiguro and Nakamura, 1994; Baumann et al., 1999; Zourelidou et al., 2002).

Another example of organ/tissue-specific expression is illustrated by a photosystem-II 10-kDa polypeptide-encoding gene (Os08g10020), which is expressed strongly in leaves and sheaths but very weakly in non-green vegetative tissues of the root, stem, mature panicle and seed (Cai et al., 2007). The promoter of this gene spans the −2,134 to +41 region, punctuated by proximal core promoter signals such as ‘*TATA-box’* and ‘*CAAT-box*’. The full-length promoter confers 14-fold, 90-fold, 14-fold and 129-fold higher expression in the leaves than in the stem, endosperm/embryo, panicle and root, respectively. As positive regulator, the ‘*leaf and panicle/stem-specific element*’ *LPSE1* (‘ATTTGAGCTGCC’) within −106 to −96 defines the expression in leaves and young panicles. As negative regulators, the ‘*leaf, panicle/stem and root element*’ *LPSRE2* (‘TTAGATAATGGA’) within −545 to −534 suppresses expression in the leaf, root, young panicle and stem, while the ‘*panicle/stem-specific element*’ *PSE1* (‘TTTATCTATTTCC’) within −708 to −696 suppresses expression in young panicle and stem. Elements with dual roles as positive and negative regulators include the *LPSRE1* (‘CGGCGCGCCAC’) within −222 to −214, *LPSE2* (‘TTGATATATTTGT’) within −383 to −371, and *PSE2* (‘TCTTTGGCAGAG’) within −670 to −659. All three elements function as enhancers in the leaf while *LPSRE1*, *LPSE2* and *PSE2* are silencers in the stem, young panicle/root, and young panicle/stem, respectively (Table 2).

#### Aging and senescence

Leaf senescence is a consequence of either developmentally programmed or stress-induced cell deterioration and declining rate of photosynthesis (Buchanan-Wollaston, 1997; Gan and Amasino, 1997; Chandlee, 2001). Cytokinin and ethylene are two major hormones that regulate the process of leaf senescence by virtue of their promoting and inhibiting effects, respectively. Other hormones and signaling molecules such as jasmonic acid, salicylic acid, abscisic acid (ABA) and reactive oxygen species also play either major or minor role singly or in combination (Nam, 1997). In rice, age-dependent leaf senescence is an important process for grain filling and seed maturation while developmentally uncoupled programmed cell death (premature senescence) is often induced by environmental stresses (Buchanan-Wolaston et al., 2003; Lim et al., 2007).

The ability to delay senescence has always been a major goal of plant biology for yield improvements by enhancing photosynthesis, i.e., ‘*stay green*’ phenotype (Thomas and Howart, 2000). Discovery of several classes of senescence-associated genes (*SAG*) provided a window to the mechanisms of senescence-regulated gene expression (Robatzek and Somssick, 2002). It was estimated that about 40 different types of transcription factors are involved in the regulation of *SAGs* including several *Myb, zinc finger, MADS-box, WRKY* and *bZIP* transcription factors (Chen et al., 2002). Knock-out mutation of senescence-associated *WRKY6* has been shown to alter the spatio-temporal expression of certain *SAGs* delaying onset of senescence.

The cysteine protease-encoding *AtSAG12* of Arabidopsis, which has a functional homolog in rice (*OsSAG39*) is highly expressed in senescing leaves, downregulated by auxin, cytokinin, and sucrose but not affected by desiccation, dark incubation, wounding, ABA or ethylene (Noh and Amasino, 1999). The senescence box or ‘*GS2*’ in the promoter of *AtSAG2* is defined by the motif ‘TTCGAAATTGAACGTGCTTAACCAAGAGAACAC’ within −603 to −571 region that binds nuclear protein extracts from senescing leaves (Table 2). Recently, it was found that the upstream region of *OsSAG39* contains positive regulatory elements within −2,021 to +24 for maximal expression when chlorophyll content is down to about 40% (Liu et al., 2010). Within its proximal and distal regions are arrays of known cis-elements such as ‘*GCCCORE*’ for ethylene and jasmonic acid regulated expression, ABA-boxes *RYREPEATBNNAPA*, *MYCATRD22* and *DPBFCOREDCDC3*, drought elements *MYBCORE* and *MYBST1*, *WRKY71OS* site for *OsWRKY71,* and elicitor responsive element *HBOXCONSENSUSPVCHS* (Urao et al., 1993; Baranowskij et al., 1994; Abe et al., 1997). *OsSAG39* promoter is inducible by the ethylene precursor ACC, ABA, GA, and low temperature. Protein extracts from senescing leaves bind to the *HBOXCONSENSUSPVCHS* and *WRKY71OS* elements (Table 2).

#### Abiotic stress and abscisic acid signaling

Drought, low temperature and salinity are essentially overlapping forms of stresses characterized by similar types of physiological perturbations and responses. At the cellular level, they trigger ‘physiological dehydration’, which affects almost every aspect of cellular physiology. As a major stress hormone in plants, the various components of abscisic acid (ABA) signal transduction pathway has been elucidated using Arabidopsis as a model (Razem et al., 2006). Seminal studies in Arabidopsis also led to the elucidation of major cis and trans components of stress response mechanisms that are evolutionarily conserved in flowering plants. Exposure to low temperature, dehydration and salinity stresses induces a common subset of genes referred to as *cor*, **co**ld **r**egulated or *rd*, **r**esponsive to **d**esiccation genes through an ABA-dependent or ABA-independent pathway (Liu et al., 1998; Thomashow, 1999; Shinozaki and Yamaguchi-Shinozaki, 2000; Yamaguchi-Shinozaki and Shinozaki, 2005).

Subsequent transduction of ABA towards gene expression involves phospholipase-D-dependent signaling and expression of various *MYB, MYC* and *bZIP* transcription factors that control the expression of different ABA responsive genes through the various combinations of cis-elements including *MybR*, *MycR* and the most predominant *ABRE* (ABA Response Element) (Finkelstein, 2006). *ABRE* interacts with bZIP-type ABRE-binding factors or ABF that function as regulators of stress response and seed maturation (Giraudat et al., 1994; Guedes Correa et al. 2008). The *ABF*-target genes include many *cor/rd* genes with the *ABRE* core motif ‘ACGT’, also referred to as *G-box* or *C-box* (Guiltinan et al., 1990). Additionally, *ABRE* functionality is combinatorial with other types of ‘non-ACGT’ coupling elements such as *CE1*, *CE3* and *DRE* (drought response element), comprising the minimal ABA-responsive complex or *ABRC* (Shen and Ho, 1995; Shen et al., 1996; Hobo et al., 1999; Narusaka et al., 2003).

In rice, the dominant ABRE-binding transcription factors have been identified as *OsABI5* and *OREB1* (Nakashima et al., 2009; Todaka et al., 2012). A recent genome-wide survey of co-expressed rice genes estimated that more than 10% (>400) of total genes are regulated through the ABA-signaling pathway, based on over-representation of the consensus sequence ‘CGMC***ACGT***B’ within −1,000 upstream regions (Cheng et al., 2007; Lenka et al., 2009; Yun et al., 2010; Table 2). Included among the ABRE-enriched genes are various components of stress signaling and defense mechanisms, further reiterating the important role of ABA as a central signal that integrates various types of stress defenses in rice (Cheng et al., 2007; Yun et al., 2010). Furthermore, genome-wide comparative analysis of ABRC composition between in rice and Arabidopsis revealed striking differences between the two species (Gomez-Porras et al., 2007; Xu et al., 2012a). While ABRCs defined by ABRE-ABRE duplex occurred at similarly high frequencies among ABA-regulated genes in both species, the occurrence of CE3 coupling element with the consensus sequence ‘(c/a)CG(c/g)(c/g)g(c/a)gC(t/g)’ is nearly specific to the ABA-responsive genes of rice (Table 2). These trends indicate that ABA-regulated genes in rice and Arabidopsis have evolved distinct combinatorial cis-element modules.

The drought, low temperature and salt-inducible expression of *cor/rd* genes in Arabidopsis requires a class of element defined by the core motif ‘(a/g)CCGAC’ named as *Dehydration Responsive Element/C-repeat* (*DRE/CRT*) (Yamaguchi-Shinozaki and Shinozaki, 1994; Table 2). This element does not function as *ABRE* but could act as a coupling element for *ABRE* to form a functional *ABRC* (Hobo et al., 1999; Yazaki et al., 2004). The trans-activators *DREB1/CBF* and *DREB2* that bind to the DRE/CRT (i.e., *DREB* for **DRE-B**inding factor or *CBF* for **C**-repeat **B**inding **F**actor) are members of the AP2/ERF family of transcription factors that are highly conserved between the dicot and monocot groups of plants (Stockinger et al., 1997; Liu et al., 1998; Stockinger et al., 2001). Constitutive expression of *DREB1/CBF* and *DREB2* have led to the coordinated activation of target *cor/rd* genes, and subsequently increased stress tolerance (Jaglo-Ottosen et al., 1998; Kasuga et al., 1999; Gilmour et al., 2000). Overexpression studies estimated that more >300 genes encoded by the Arabidopsis genome are regulated through the DREB/CBF pathway with >100 genes confirmed as direct targets (Seki et al., 2001; Fowler and Thomashow, 2002).

The DREB/CBF regulon is highly conserved across the temperate and tropical groups of flowering plants as evidenced by the occurrence of functional *DREB1/CBF* and *DREB2* orthologs in both monocots (including rice) and dicots (Jaglo et al., 2001; Choi et al., 2002; Dubouzet et al., 2003; Nakashima et al., 2009; Yun et al., 2010; Todaka et al., 2012). Genome-wide survey of the enrichment of ‘(a/g)CCGAC’ core motifs with correct spatial distribution revealed that *DRE/CRT* are as ubiquitous among the stress-regulated genes encoded by the rice genome as *ABRE*, i.e., significantly enriched in >10% of total rice genes, similar to what has been observed in Arabidopsis (Rabbani et al., 2003; Fowler and Thomashow, 2002; Yazaki et al., 2004; Vogel et al., 2005; Cheng et al., 2007; Lenka et al., 2009). For example, *DRE/CRT* is the dominant class of cis-elements that regulates a late embryogenesis abundant protein (*OsLEA*) network involved in dehydration, low temperature and salinity stress mechanisms in japonica rice (Meier et al., 2008).

The promoter of Arabidopsis *CBF/DREB* (e.g., *CBF3)* is characterized by a highly conserved 125-bp module as binding sites (ICE Boxes I, II, III, IV, V) for a Myc-like bHLH transcription factor *ICE1* (Shinwari et al., 1998; Chinnusamy et al., 2003; Zarka et al., 2003). *ICE1* is regulated by phosphorylation and its activated form binds to the *c-Myc* target element (‘CANNTG’) located in the highly conserved 125-bp region of *CBF* promoters. Box IV within the critical 125-bp region of *CBF2* promoter contains the motif ‘CACATG’. Similar survey of upstream regions of all members of the orthologous *DREB/CBF* gene families in rice and sorghum revealed common signatures that include cis-elements like Ca/Calmodulin element (‘VCGCGB’), *ABRE* (‘CGTGG’, ‘MACGYGB’), jasmonic acid signaling (‘CGTCA’, ‘TGACG’) and MYC (‘CANNTG’) (Srivastav et al., 2010).

While the DREB/CBF network appears to play the dominant role low temperature (4°C and below) and drought response mechanisms in rice, several recent studies revealed other regulons with likely overlap with biotic stress response networks through oxidative signaling (Table 2). An example is the NAC regulon controlled by the *NAC*-type (*NAM, ATAF, CUC*) transcription factors *OsNAC6/SNAC2* and *OsNAC5*, with target cis-elements having the core motif ‘CACG’ (Nakashima et al., 2007; Hu et al., 2008; Nakashima et al., 2009). Another example is the network of genes regulated by bZIP transcription factor *OsTGA10* (Os06g41100) and R2R3-type Myb transcription factor *OsMyb4* (Os04g43680), which is activated primarily in by oxidative signal stress during exposure to chilling and configures various components of oxidative defenses (Jakoby et al., 2002; Cheng et al., 2007; Park et al., 2010b; Yun et al., 2010; Xu et al., 2012). This network is comprised of a large number of target genes defined by several combinations of *as1/ocs/TGA*-like elements (‘AATTTGAT’, ‘TAATTTGA’) and *GARE/Pyr-box/Myb2-box*-like elements (‘AAAGAAAAA’, ‘TAGTTTTT’).

#### Biotic stress and ethylene, salicylic acid and jasmonic acid signaling

Ethylene, salicylic acid and jasmonic acid are key components of host plant signaling and defenses against pathogens and insect herbivores (Abeles et al. 1992; Pieterse et al., 1998; Johansson et al., 2006; Dempsey and Klessig, 2012). Ethylene signaling during host-pathogen or host-herbivore interactions lead to changes in gene expression through various types of ERF (Ethylene Response Factor) transcription factors, while the salicylic acid and jasmonic acid mediated gene expression involves the action of various types of WRKY and bHLH transcription factors, respectively (Helliwell and Yang 2013). For instance, the early responses of rice against the striped stemborer *Chilo suppressalis* is known to involve ethylene, salicylic acid, jasmonic acid and H_2_O_2_, mediating a MAPK-phosphorylation cascade that regulates the expression of several *OsWRKY* and *OsERF3* transcription factors (Lu et al., 2011). Similarly, the early responses of rice to both virulent and avirulent pathovars of *Magnaporthe oryzae* (blast) is mediated by ethylene leading to the upregulation of *OsERF922* transcription factor and its suite of target defense-related genes (Singh et al., 2004; Liu et al., 2012).

Examples of known targets of defense-related ERF transcription factors are the pathogenesis-related proteins *PR3*, *PR4* and *PDF1.2*. A common feature shared by many of these PR-protein genes and other types of ethylene-regulated defense genes is the canonical *GCC-box* cis-element defined by the core motif ‘AGCCGCC’ (Hart et al., 1993; Ohme-Takagi and Shinshi, 1995; Manners et al., 1998; Suzuki et al., 1998; Table 2). A more recent example of ERF-type transcription factors that directly target defense-related genes in rice is *OsERF922*, which acts on *GCC-box* containing genes to negatively regulate resistance to *Magnaporthe oryzae* (Liu et al., 2012). In Arabidopsis, other types of ERF transcription factors such as *Pti4/5/6* have also been shown to bind to the canonical *GCC-box* as well as other *GCC-box* related core-motifs such as ‘TAAGAGCCGCC’ (Table 2). These ERF transcription factors and their cognate cis-elements are highly conserved in both dicot and monocot plants including rice (Hao et al., 1998; Gu et al., 2000). Additionally, the conserved ‘*GCC-box*’ has also been shown to function as JA-responsive cis-element involved in both pathogen and herbivore defense mechanisms in Arabidopsis and rice (Brown et al., 2003). Many such ERF-type transcription factors that act on ‘*GCC-box*’ cis-element have been shown to modulate the expression of their target ethylene-responsive and jasmonic acid-responsive genes either positively or negatively, by virtue of their dual activation and repression domains (Rojo et al.*,*^1999^; Fujimoto et al., 2000; Ohta et al., 2001; Ohme-Takagi et al., 2000; Bodenhausen and Reymond, 2007).

Recent studies revealed other defense-related ERF transcription factors that regulate ethylene-inducible gene expression through the *P-box* element, defined by the core motif ‘CCGCCCTCCA’ (Table 2). Initially identified in the promoter of ethylene and pathogen-inducible putrescine-N-methyltransferase-2 (*PMT2*) gene of tobacco, *P-box* element binds various ERF- transcription factors that are not involved in GCC-box-mediated gene expression (e.g., *ERF189*, *ERF115*, *ERF179*, *ERF163*, *ORCA3*, *AtERF13). P-box*-like motifs have also been shown to be highly enriched among putative ERF-target defense-related genes in rice (Shoji et al., 2010; Shoji and Hashimoto 2012).

Salicylic acid plays a major role in defense mechanisms particularly against biotrophic pathogens that parasitize a living host (Alvarez, 2000; Desveaux et al., 2004; Garcion and Métraux, 2006; Ciolkowski et al., 2008; Vlot et al., 2009). In rice, salicylic acid-mediated gene expression has been shown to involve similar types of cis-elements and transcriptional regulators that have been identified in other dicot plants such as Arabidopsis and tobacco (Silverman et al., 1995; Yang *et al*., 2004). Primarily, salicylic acid-mediated defense involves WRKY transcription factors acting on the *SA-responsive* element defined by the core motif ‘TGACG’ and *W-box* element defined by the core motif ‘TTGAC(c/t)’ (Chen et al., 2002; Li et al., 2004). In rice, several WRKY transcription factors (*OsWRKY45*, *OsWRKY71*, *OsWRKY03*) have been shown to bind to *W-box* of several PR-protein genes such as *OsPR10a* (Liu et al. 2005b; Shimono et al. 2007; Hwang et al. 2008; Table 2).

Many members of WRKY family of transcription factors in rice are transcriptionally activated in response to *M. oryzae,* the causal agent of blast and *Xanthomonas oryzae pv. oryzae*, the causal agent of bacterial leaf blight (Ryu *et al.*, 2006; Bagnaresi *et al.*, 2012).

For example, *OsWRKY45* has been shown to play a dual role in mechanisms of resistance to both blast and bacterial leaf blight, while many other WRKY transcription factors such as *OsWRKY53*, *OsWRKY31*, and *OsWRKY30* have been shown to function specifically as positive regulators of defenses against blast by virtue of their ability to activate a suite of target genes with common features of having either *W-box* and/or *SA-response* elements (Zhang et al., 2008; Peng et al., 2012; Shimono et al.*,*^2012^). Some WRKY transcription factors such as *OsWRKY76* function as negative regulators of disease resistance also by virtue of their interaction with the *W-box*. This indicates that the *W-box* functions both as activator and repressor of SA-mediated gene expression in rice (Yokotani et al. 2013).

Jasmonic acid is an important regulator of responses to pathogens, insect herbivores and other related factors such as wounding and mechanical injury (Creelman and Mullet, 1995; Farmer et al., 2003; Wasternack, 2007; Koo and Howe, 2009). The primary activators of jasmonic acid-mediated signaling are bHLH-type transcription factors that are conserved between Arabidopsis and rice. The precise identity of the associated activator and repressor cis-elements for jasmonic acid-mediated gene expression are just starting to be revealed, but a number of candidates have recently been suggested. For example, three copies of an *E-box* element defined by the core motif ‘CANNTG’ has been suggested to function as JA-responsive enhancer within −515 to −265 region of rice chitinase gene *OsChia4a* (Table 2). The *E-box* element has been suggested as the binding site for the bHLH-type *RERJ1* transcription factor (Nishizawa et al., 1999; Miyamoto et al., 2012; Wager and Browse 2012; Santino et al., 2013). Recently, another bHLH-type transcription factor *OsbHLH65* that responds to *Magnaporthe grisea*, *Nilaparvata lugens,* salicylic acid and jasmonic acid directly binds to several potential *E-boxes* with the signature motifs ‘CAGCTG’ and ‘CACCTG’ (Santino et al., 2013).

#### Cis-element and transcription factor databases

Systematic curation of cis-elements and their cognate trans-acting factors that define regulated transcription in eukaryotes is central to computational modelling of transcriptional networks (Buchler et al., 2003; Xie et al., 2005; Mohanty et al., 2012). Our recent survey of regulatory sequences of rice genes across the available public databases indicate that rice information has been steadily increasing given the level of global activity in rice functional genomics (Tables 2 and 3). During the last two decades, several data repositories have been developed to provide an annotated reference of cis-elements and their cognate trans-acting factors established from the results of wet-lab experiments. The first generation information repositories which include *TRANSFAC*, *PLACE*, *PlantCARE* and *AGRIS* served as initial foundation for large-scale computational prediction of the unifying regulatory features among co-expressed genes revealed by genome-wide transcriptome studies (Wingender et al., 1996; Higo et al., 1999; Lescot et al., 2002; Davuluri et al., 2003; Matys et al., 2003). However, while *TRANSFAC* has been utilized for the analysis of rice data, its predictive power for novel plant-specific elements is quite limited. On the other hand, *PLACE*, *PlantCARE* and *AGRIS* have offered relatively higher resolutions for the analysis of rice data, given their stronger emphasis on monocot and dicot plants.

**Table 3:** **Partial list of recently annotated regulatory sequences from rice based on recent updates in public cis-element and transcription factor databases**

**Cis-element**	**Consensus core motif**	**Transcriptional regulator**	**Biological function**
***AACA element***	AACAAAC	*OsMYB5*	Regulator of endosperm-specific expression
***ABRE***	GTACGTGGCGC	Unknown	Positive regulator of GA response; Negative regulator of ABA response
	ACGTGKC	Unknown	ABA-response element for ABA regulated gene expression
	GCCGCGTGGC	Unknown	ABA-response element for ABA regulated gene expression
	AGTACGTGGC	Unknown	ABA-response element for ABA regulated gene expression
***ABRE/hex-3***	GGACGCGTGGC	*OsABF1*	ABA-response element for ABA regulated gene expression
***ABRE-like***	TACGTGTC	*OsTRAB1*	ABA-response element for ABA regulated gene expression
***G-box***	RTACGTGGCR	*OsTAF-1*	Positive regulator for response to ABA and desiccation in vegetative tissues
	CTTCCACGTGGCA	*OsBZ8*	ABA-response element for ABA regulated gene expression
***RY repeat***	CATGCATG	Unknown	ABA-response element for ABA regulated gene expression
***AH1 element***	CAATTATTG	*OsHox1*	Enhancer of provascular-specific expression
***ARR1 element***	AGATT	*OsARR1*	Non-symbiotic hemoglobin-2 promoter
***as1-like***	GCATCTTTACTTTAGCATC	as1-like box binding factor	Phloem-specific expression
***IIb element***	TGTGGGACCATG	*OsPCF1, OsPCF2*	Enhancer of meristem-specific expression
***BIHD1OS element***	TGTCA	*OsBIHD1*	Pathogen-regulated expression
***BP-73 element***	AACGT	*OsBP-73*	Regulation of cell proliferation
***E2F element***	GCGGGAAA	*OsE2F*	Enhancer in actively dividing cells
***GATA box***	GATA	*OsASF-2*	Light regulated expression
***GCC-box***	AGCCGCC	*OsEREBP1*	Pathogen-regulated expression
	CATAAGAGCCGCCACT	*OsBIERF3*	Response to biotic and abiotic stress
***IDE1 element***	ATCAAGCATGCTTCTTGC	*OsIDE1, OsIDE2*	Iron deficiency regulated expression
***OsIRO2 element***	CACGTGG	*OsIRO2*	Iron deficiency regulated expression
***PR2 element***	ACGCTGCCG	*OsWOX9*	Root meristem-specific expression
***TGTCA element***	TGTCA	*OsBIHD1*	Pathogen-regulated expression
***W-box***	TTGAC	*OsWRKY13, OsWRKY51, OsWRKY71*	Pathogen-regulated expression

More recent generations of cis-element databases like the Database of Arabidopsis Transcription Factors (*DATF*), Database of Rice Transcription Factor (*DRTF*), Plant Transcription Factor Database (*Plant TFDB*), and Arabidopsis Stress Responsive Transcription Factor Database (*STIFDB*) have significantly improved the resolution of regulatory sequence prediction in rice genomic sequences given the broader emphasis on plant physiological processes (Guo et al., 2005; Gao et al., 2006; Riaño-Pachón et al., 2007; Shameer et al., 2009; Perez-Rodriguez et al., 2010; Wang et al., 2010; Zhang et al., 2011). *DRTF* in particular holds information on more than 2,000 putative transcription factors and their computationally predicted target cis-elements anchored to the reference genome sequence of rice. Other more recent additions to available databases include the Plant Transcription Factor database (*PlantTFDB)***,***GRASSIUS* and *Osiris* (Guo et al., 2008; Morris et al., 2008; Yilmaz et al., 2009; Zhang et al., 2011).

In summary, the number of public databases that curate information about regulatory sequences and transcription factors in plants has increased substantially over the last decade. The information contained in these resources are evolving constantly and their maximum utility for high-resolution computational prediction of transcriptional networks in rice relies on systematic annotation based on experimentally validated data. What is currently necessary for more biologically meaningful analysis of regulatory sequence variation in the genus *Oryza* is a systematic means to integrate and synthesize the data from all resources in order to resolve inconsistencies in annotation, redundancy, and lack of uniform nomenclature.

## Conclusions and perspectives

### Applications of upstream sequence variation to genus-wide gene discovery, allele mining and network engineering

The fundamental question about the architecture of rice promoters and the nature of their cognate regulator proteins remains very relevant in the era of comparative functional genomics. Our recent survey of the major public eukaryotic cis-element and transcription factor databases have indicated that sequences and annotation of cis-elements and their cognate transcriptional regulators in the reference rice genome are still far from being comprehensive to guide a systematic scrutiny of regulatory networks at a genome-wide and comparative scales. The ‘finished’ reference genome sequence of rice has been available to the community now for a decade, serving as nucleator for hypothesis-driven interrogation of gene function and evolution. As we enter the next decade of reference genome-enabled experimentation in rice genetics, which has directed its focus on genotypic comparisons, allele and novel gene mining, the importance of a comprehensive annotation of cis-acting elements and trans-acting factors that define regulatory networks cannot be over-emphasized.

**Figure 3:**
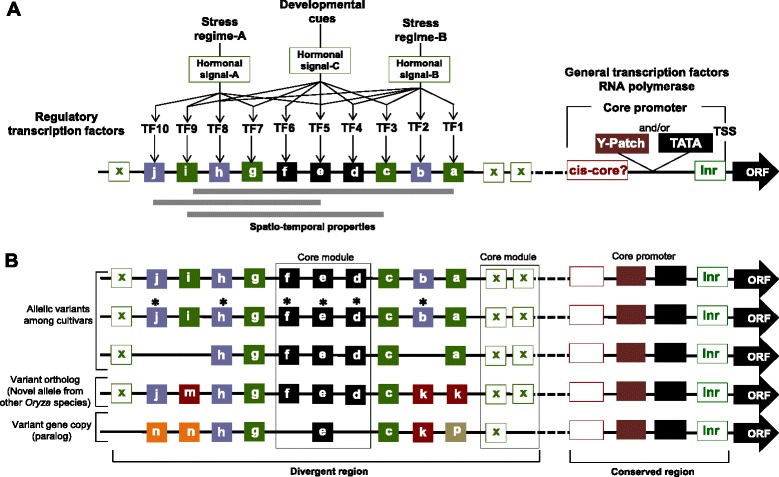
**Hypothetical and simplistic models of upstream regulatory information content of spatio-temporally regulated genes of rice.** **(A)** Prototype inducible promoter is comprised of combinations of cis-elements (*i.e., colored boxes upstream from the core promoter identified by lowercase letters*) that directly interface gene induction/represssion with various environmental and developmental signals. This occurs by virtue of their respective cognate regulatory transcription factors (*i.e., TF1, TF2, etc.*) that respond to hormonal or other physico-chemical changes in the cell. The spatio-temporal properties of genes are therefore defined by the integration of external and developmental cues through the synergistic interactions of various cis-elements and their cognate regulatory transcription factors. **(B)** Example of a pattern/trend that could be revealed by phylogenetic footprinting of upstream regulatory sequences of rice genes with potential significance to comparative functional genomics and allele mining. Phylogenetic footprinting has suggested that functional differences between the promoters of genes that vary in spatio-temporal regulation may be due to combinations of divergent regulatory fine-tuner elements (*colored squares*) and highly conserved core module elements (*black squares*). Variation in cis-regulatory information content may therefore reveal the genomic basis for variant alleles with unique spatio-temporal properties. This concept may be extrapolated for interspecific comparisons of orthologs and paralogs towards the discovery of potential novel alleles in the genus *Oryza*. Differences in expression in the absence of apparent upstream sequence variation may be used as basis for targeted analysis of differential methylation (shown with asterisks*) of homologous promoters. This concept may have potential applications for the discovery of epialleles that determine phenotypic variation.

Creating novel combinations of superior alleles by integrative use of conventional and biotechnological methods is the overarching goal of 21^st^ century rice breeding. The genome-enabled research paradigm in rice biology has contributed to this goal through the understanding of gene function and regulation. Moreover, at the very core of the reference genome-guided research paradigm is the quest to pinpoint useful allelic variation in the germplasm that explains simple or quantitative traits to guide targeted introgression and selection. Broadly, gene function can be described in the context of a gene’s specific molecular, biochemical and/or biological roles in the cell and in context of its role in a broader molecular or biochemical network. The upstream regulatory sequence signature of a gene is a window to the combinatorial complexity of its regulation and reflects how it is interfaced with the various intrinsic and extrinsic signals for growth, development, reproduction and adaptation (Figure 3A). In these contexts, a truly biologically meaningful annotation of the reference genomes of rice that could facilitate a holistic understanding of the nature of allelic and epiallelic variation for phenotypes of interest must include information on the critical cis-elements and trans-acting factors that define the spatio-temporal regulatory properties of each gene. Establishing a modular map of regulatory elements within the upstream regions of each gene locus as part of reference genome annotations in a similar manner that we understand protein domain architectures of coding sequences will be an important tool for various applications in comparative functional genomics both at the intraspecific and interspecific levels.

The specific applications of upstream sequence annotation are numerous and some of the more important concepts are discussed in this review. First, in-depth understanding of the modular architectures of upstream regulatory regions for each annotated gene locus (i.e., open-reading frame) in the reference genome could nucleate direct comparison of orthologs and paralogs by phylogenetic footprinting across a meaningful germplasm diversity panel. Phylogenetic footprinting is based on the assumption that conserved sequence motifs or modular combinations of motifs within the upstream sequences of homologous genes represent functional cis-elements that define the spatio-temporal regulation of that gene (Hardison, 2000; Hong et al., 2003; Zhang and Gerstein, 2003; De Bodt et al., 2006; Freeling and Subramaniam, 2009; Xu et al., 2012a). In other words, patterns of cis-element conservation or divergence revealed by phylogenetic footprints define the integrated effects of various developmental and external signals on the expression potential of a given gene (Figure 3A). An offshoot of that concept is that upstream sequences containing critical regulatory elements can be the subject of targeted scrutiny to reveal meaningful sequence variation both at the intraspecific and interspecific levels for the purpose of uncovering conserved or divergent patterns associated with expression variability and elite phenotypes (Figure 3B). This is an area in rice comparative functional genomics that has not been actively pursued given the rapidly growing resources for comparative functional genomics for mining novel alleles. As more germplasm resequencing data become available, meaningful comparative analysis of upstream regulatory sequence variation by phylogenetic footprinting must be pursued towards the discovery of novel alleles and associated networks with potential applications in rice breeding.

An example of a seminal study that highlights the potential of phylogenetic footprinting for novel gene discovery and allele mining was a recent comparison of upstream regulatory sequence signatures across orthologous and paralogous groups of stress-associated bZIP transcription factors of rice, Arabidopsis and sorghum, three species representing more than 140 million years of evolutionary history and unique geo-climatic distribution (Xu et al., 2012a). It was revealed that while orthologs shared very similar basal developmental programming by virtue of highly conserved ‘*core modules*’ of cis-elements, they also exhibit unique cis-element footprints hypothesized to act as ‘*regulatory fine tuners*’ in conjunction with the ‘*core modules*’. It was inferred that differences in upstream sequence footprints contributed by the ‘*regulatory fine tuners*’ may serve to highlight the unique spatio-temporal characteristics of orthologs and paralogs, underscoring the potential uniqueness of each ortholog and paralog in a regulatory context hence expression variability. Indeed, the trends established from such comparison has been supported by similar patterns established based on phylogenetic footprints of upstream non-coding sequences between rice and other syntenic cereal genomes including maize, sorghum and barley, suggesting that phylogenetic footprints could be a powerful tool for revealing some of the major determinants of novel allelic variation (Guo and Moose, 2003; Wang et al., 2009).

The genus *Oryza* represents about 15 million years of evolutionary divergence that has been established as a ‘*one-of-its-kind*’ tractable comparative genomics system. Capitalizing on the ‘finished’ reference sequence of *O. sativa* ssp. japonica, an international consortium is currently developing a genus-wide platform for the discovery of novel genes and for mining novel alleles with potential use in genomics-enabled introgression breeding (Wing et al., 2005; Jacquemin et al., 2012). To this date, a comprehensive set of sequence scaffolds representing all diploid genomes (i.e., AA, BB, CC, EE, FF, and GG) has been developed and in various stage of sequence assembly and completion, with few already released including the African cultivated species *O. glaberrima* (AA) and the wild species *O. barthii* (AA), *O. longistaminata* (AA), *O. nivara* (AA), *O. punctata* (AA), *O. rufipogon* (AA), and *O. brachyantha* (FF) (Rounsley et al., 2009; Xu et al., 2012b; Chen et al., 2013; Jacquemin et al., 2014). Given this rapidly unfolding genomics resources, it would soon be possible to conduct a meaningful global and cross-genomic analysis of orthologous and paralogous upstream sequence and expression variation in *Oryza*. The resulting knowledge may provide an important window to the nature of allelic variation for rice genes that are regulated at the transcriptional level, and could set the stage for targeted gene introgression and allele replacement in the future.

Second, it is important to articulate that the true essence of functional genomics in the context of rice breeding must be deeply rooted to the ability to identify meaningful sequence variation across an allelic series, understanding how such sequence variation contributes to differences in gene function, and whether differences in gene function are due simply to sequence variation (alleles) or differential DNA methylation in the absence of drastic sequence variation (epialleles). More and more examples of homologous genes that vary in expression in the absence of meaningful sequence variation are being reported in the literature (Peng and Zhang, 2009; Hai et al., 2013; Zheng et al., 2013). Understanding the organization and distribution of critical regulatory element modules within the upstream sequences of intraspecific or interspecific homologs has the potential to provide another layer of information for understanding the contribution of ‘*epiallelic variation*’ to phenotypic variation. In this context, homologous genes that differ in expression characteristics despite the absence of any meaningful differences in upstream regulatory sequence architecture should justify examination of the contribution of differential upstream DNA methylation in the expression of the desired phenotype (Figure 3B). While the concept of epiallelic introgression to phenotypic gain is still vague and yet to be explored in rice genetics and breeding, the establishment of reference genome sequences with well annotated upstream regulatory sequences should set the stage for future large-scale association between DNA methylation patterns and upstream sequence variation. Certainly, the rapidly unfolding reference genome sequence resources across the genus Oryza will serve as foundation for the discovery of potential epiallelic series that are due to differential upstream sequence methylation.

Third, it is well established that the major transcriptional regulatory networks involved in stress response mechanisms are highly conserved or universal in flowering plants. For instance, the *DREB/CBF* regulon has functioning equivalents in plant species with contrasting sensitivities to low temperature such as rice (intolerant) and Arabidopsis (tolerant), or to drought such as rice (intolerant) and sorghum (tolerant). *DREB/CBF* regulons are also equally functional even between any two rice genotypes that differ significantly in regard to relative stress tolerance. What remains unclear is how variation in the activity of the *DREB/CBF* regulatory network contributes to phenotypic gradient for stress tolerance at the intraspecific and interspecific levels. One theory is that regulatory networks like the *DREB/CBF* regulon may contribute to phenotypic gradient by virtue of variation in size and compositional complexity of downstream genes that are controlled by the master regulator. It has been proposed that this may be dependent on the relative distribution of the target cis-elements across individual genomes (Yun et al., 2010; Zhang et al., 2004). Testing this hypothesis in rice will require a systematic survey of genome-wide distribution of *bona-fide DREB/CBF* target cis-element modules across a meaningful diversity panel that represents the gradient of stress sensitivity in the germplasm.

The outcome of such type of analysis could also contribute to the understanding of how regulatory networks can be reconfigured by altering the distribution of critical cis-regulatory modules by homologous recombination and genome shuffling during the process of parental intercrossing. This concept could also lead to future exploration of the potential of transcription factor and target gene cluster complementation during introgression. For instance, a novel transcription factor homolog from a donor may be combined with a much more elaborate array of downstream target genes that contain the critical cis-element in the recipient genome. The possible effect could be the further elaboration of the regulon hence novel configuration of the transcriptional network due to such novel combination of master switch and downstream target cis-elements. This may lead to physiological reconfiguration and may be explored as foundation for the creation of novel and/or transgressive phenotypes by breeding.

Finally, one major application of understanding cis-element function that has been explored since the early days of rice biotechnology is the concept of ‘*designer promoters*’ (Mehrotra et al., 2011). Given the growing comparative genomics resources in rice, this concept may be revisited with a slightly different set of questions. The classical concept asked the question regarding the feasibility of finely optimizing the spatio-temporal expression of a regulon that defines the expression of a complex phenotype such as drought tolerance by driving its major regulatory gene (i.e., regulatory hub) under the control of a novel and ideal chimeric promoter. Conversely in today’s context, given the emerging technology for gene editing and replacement, it may be relevant to ask the question on the feasibility of using our knowledge of cis-element architecture to alter the distribution of critical cis-elements in the genome to create a more elaborate transcriptional regulatory network and novel phenotypes. These are some of the forward-looking questions in rice regulon engineering that would require in-depth understanding of the nature and distribution cis-elements and their cognate regulators in a genome-wide scale. The rapid progress in rice genetics facilitated by reference sequence-enabled comparative genomics in *Oryza* should nucleate future studies addressing some of these concepts.

## Abbreviations

TSS: Transcription start site
TBP: TATA-binding protein
BRE: TFIIB recognition element
DPE: Downstream promoter element
IME: Intron-mediated enhancement
IAA: Indole 3-acetic acid or auxin
ABA: Abscisic acid
GA: Gibberellic acid
SRC: Sugar response complex
JA: Jasmonic acid
SA: Salicylic acid
